# Draft Genome Sequence of *Chromobacterium haemolyticum* Causing Human Bacteremia Infection in Japan

**DOI:** 10.1128/genomeA.01047-14

**Published:** 2014-11-06

**Authors:** Tsuyoshi Miki, Nobuhiko Okada

**Affiliations:** Department of Microbiology, School of Pharmacy, Kitasato University, Tokyo, Japan

## Abstract

*Chromobacterium haemolyticum* is a Gram-negative bacterium displaying remarkable hemolysis against human and sheep erythrocytes. In addition, *C. haemolyticum* infects humans, in which the infection mechanism remains unknown. We report here the draft genome sequence of *C. haemolyticum* strain T124, isolated from a young patient with sepsis in Japan.

## GENOME ANNOUNCEMENT

*Chromobacterium haemolyticum* is a Gram-negative bacterium that has a remarkable ability to lyse human and sheep erythrocytes (1). The genus *Chromobacterium* consists of seven species, including *C. violaceum* and *C. haemolyticum*. *C. violaceum* is an environmental bacterium living especially in tropical regions and has also been recognized as a pathogenic bacterium. Although rare, an infection with *C. violaceum* progresses rapidly to various organs, especially the lungs, liver, and spleen, and thus is a life-threatening sepsis having highly mortality rates (2, 3). The genome sequence of *C. violaceum* revealed the presence of potential virulence genes, especially three gene clusters encoding two type III secretion systems (T3SSs) (4, 5). The T3SS is a common virulence determinant in various Gram-negative pathogenic bacteria by injecting bacterial toxins, called effectors, into the host cell directly. We previously showed that a T3SS encoded by *Chromobacterium* pathogenicity island 1 and 1a (Cpi-1/-1a) is a major virulence determinant (6) and that an effector CopE secreted from the Cpi-1/1a–encoded secretion machinery plays a critical role in host cell invasion and systemic infection in mice (7). Recently, clinical evidence for invasive infection with *C. haemolyticum* has been reported in Japan (8). At present, infection mechanisms by the human pathogen *C. haemolyticum* remain completely unknown. Here, we briefly announce the draft genome sequence of one clinical isolate of *C. haemolyticum* strain T124, which will provide novel insight into and a better understanding of infections with this bacterium.

The whole genome of *C. haemolyticum* T124 was sequenced using a combined Illumina HiSeq 2000 and Roche GS-FLX approach. Reads obtained from sequencing by Illumina HiSeq were assembled using Velvet. Furthermore, reads obtained from sequencing by Roche GS-FLX were combined with the passed Illumina reads and then were assembled using the Genome Sequencer *de novo* assembler. The hybrid data yielded 72 large contigs (≥500 bp each) with *N*_50_ contig sizes of 189,800 bp, with an average length of 70,602 bp and a largest length of 745,057 bp. The resulting genome sequence of *C. haemolyticum* T124 is 5,110,775 bp with 161-fold coverage and has a G+C content of 62.8%. Annotation by the Rapid Annotations using Subsystems Technology (RAST) sever revealed 4,557 coding genes, 73 tRNA genes, and 8 copies of the rRNA genes. In addition, RAST showed that the closest neighbor is *C. violaceum* ATCC 12472 (4).

Similar to *C. violaceum* ATCC 12472, *C. haemolyticum* T124 contains the virulence-associated type III secretion gene cluster. ATCC 12472 has two distinct T3SSs encoded by Cpi-1/-1a and -2, respectively, whereas T124 lacks the Cpi-2-like second T3SS. These data suggest that a major virulence determinant of *C. haemolyticum* T124 might be the Cpi-1/-1a–like T3SS. The genome sequence reported here will aid in future studies of *C. haemolyticum* to understand the fatal infection mechanism and to develop efficient antimicrobial therapy against the life-threatening sepsis.

### Nucleotide sequence accession numbers.

This whole-genome shotgun project has been deposited at DDBJ/EMBL/GenBank under the accession number JRFR00000000. The version described in this paper is version JRFR01000000.
